# Supplementary material for: DNA copy number motifs are strong and independent predictors of survival in breast cancer
Source: Commun Biol. 2020 Apr 2;3:153. doi: 10.1038/s42003-020-0884-6 (PMC7118095; doi:10.1038/s42003-020-0884-6)
Supplement: Supplementary file 1 — Supplementary Information [file 42003_2020_884_MOESM1_ESM.pdf]

Supplementary Figures

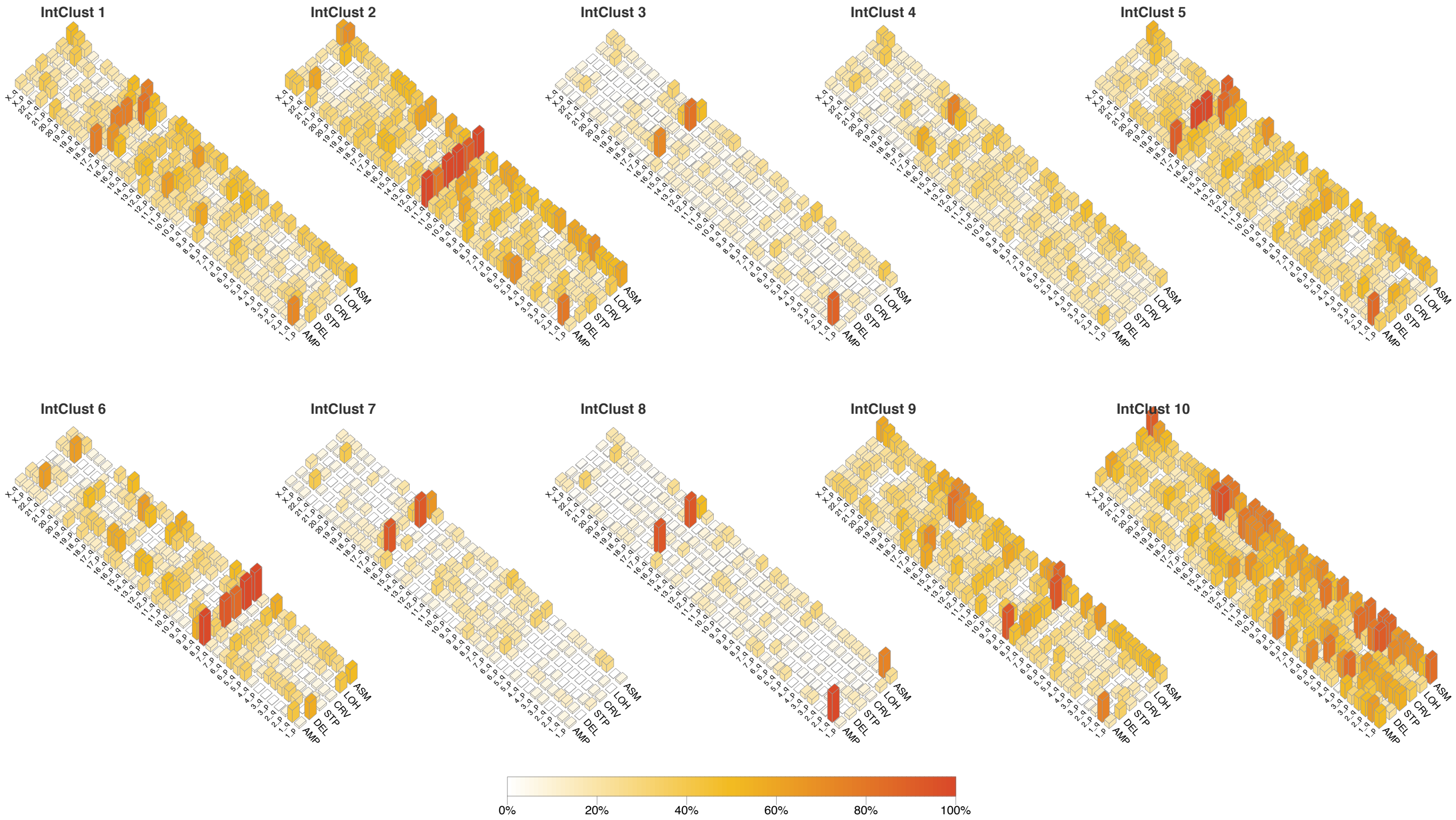

Supplementary Figure 1. Score landscapes for the IntClust subtypes in the Oslo2 cohort. The bars reflect, for each of the six CARMA indices and each chromosome arm, the percentage of tumors in the Oslo2 cohort that have a score larger than the index median (calculated across all arms and ignoring zero scores within each of the CARMA indices in the METABRIC discovery set). See Supplementary Table 1 for the number of tumors in each IntClust subtype.

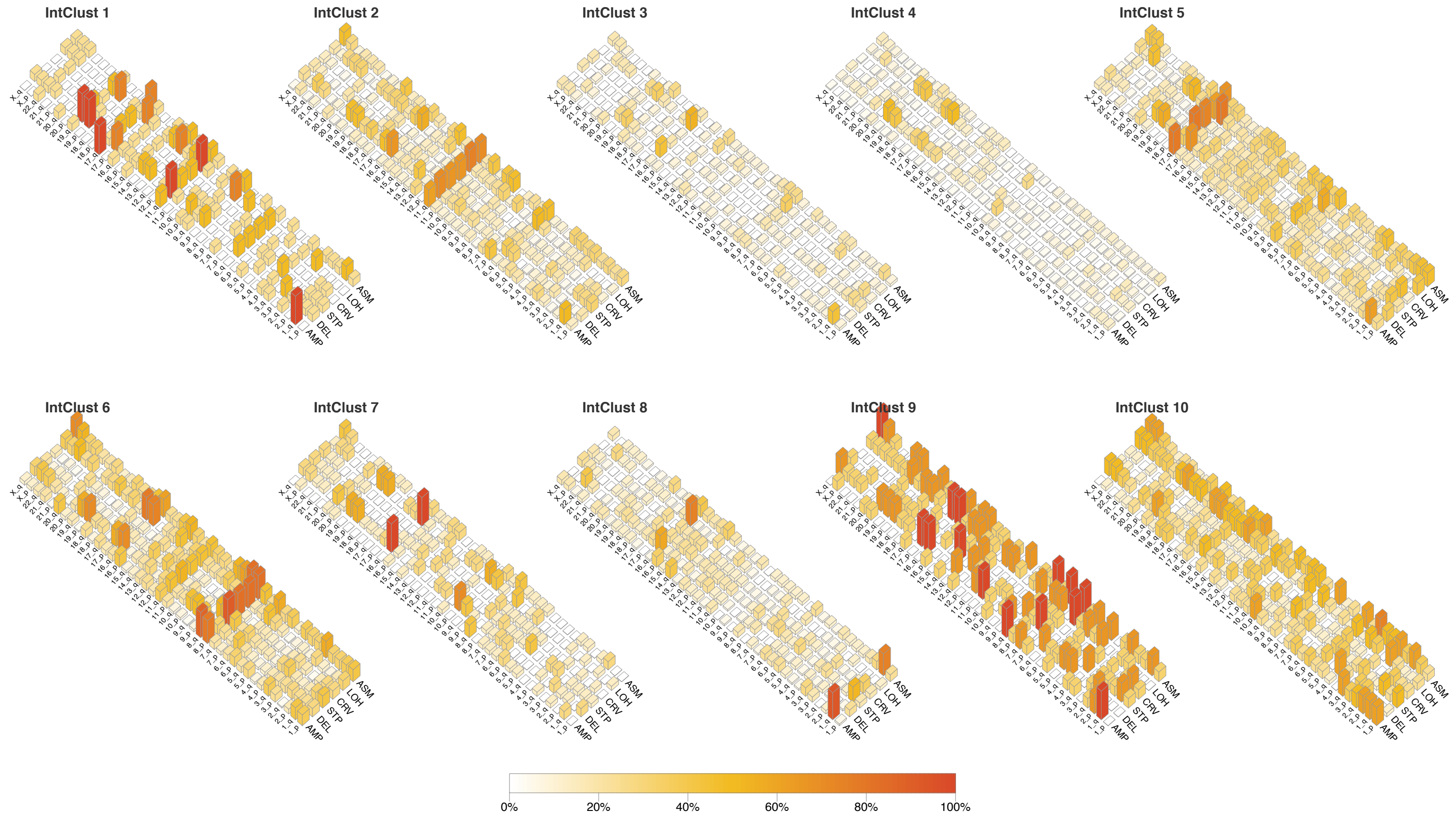

Supplementary Figure 2. Score landscapes for the IntClust subtypes in the OsloVal cohort. The bars reflect, for each of the six CARMA indices and each chromosome arm, the percentage of tumors in the OsloVal cohort that have a score larger than the index median (calculated across all arms and ignoring zero scores within each of the CARMA indices in the METABRIC discovery set). See Supplementary Table 1 for the number of tumors in each IntClust subtype.

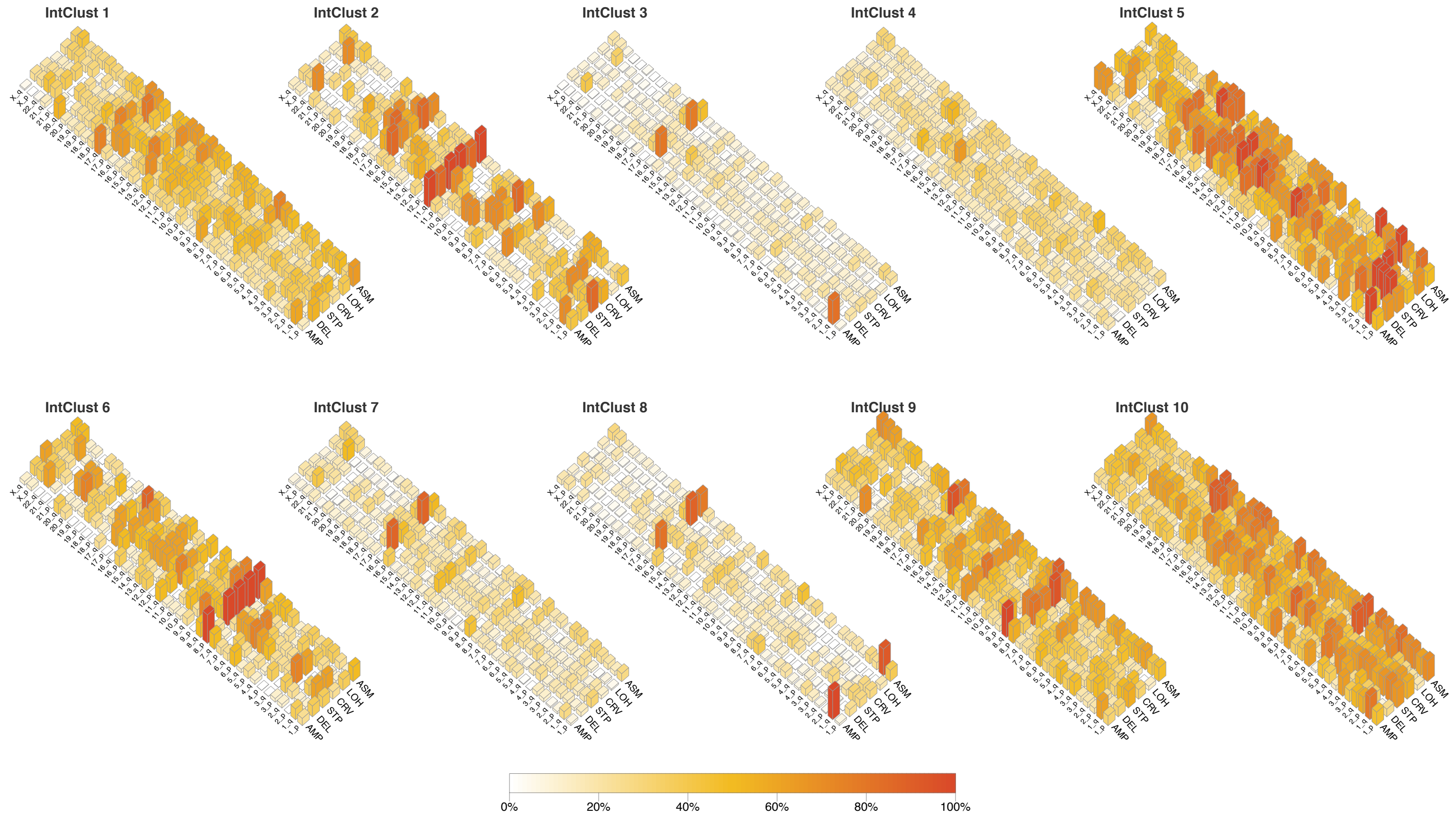

Supplementary Figure 3. Score landscapes for the IntClust subtypes in the ICGC-BRCA cohort. The bars reflect, for each of the six CARMA indices and each chromosome arm, the percentage of tumors in the ICGC-BRCA cohort that have a score larger than the index median (calculated across all arms and ignoring zero scores within each of the CARMA indices in the METABRIC discovery set). See Supplementary Table 1 for the number of tumors in each IntClust subtype.

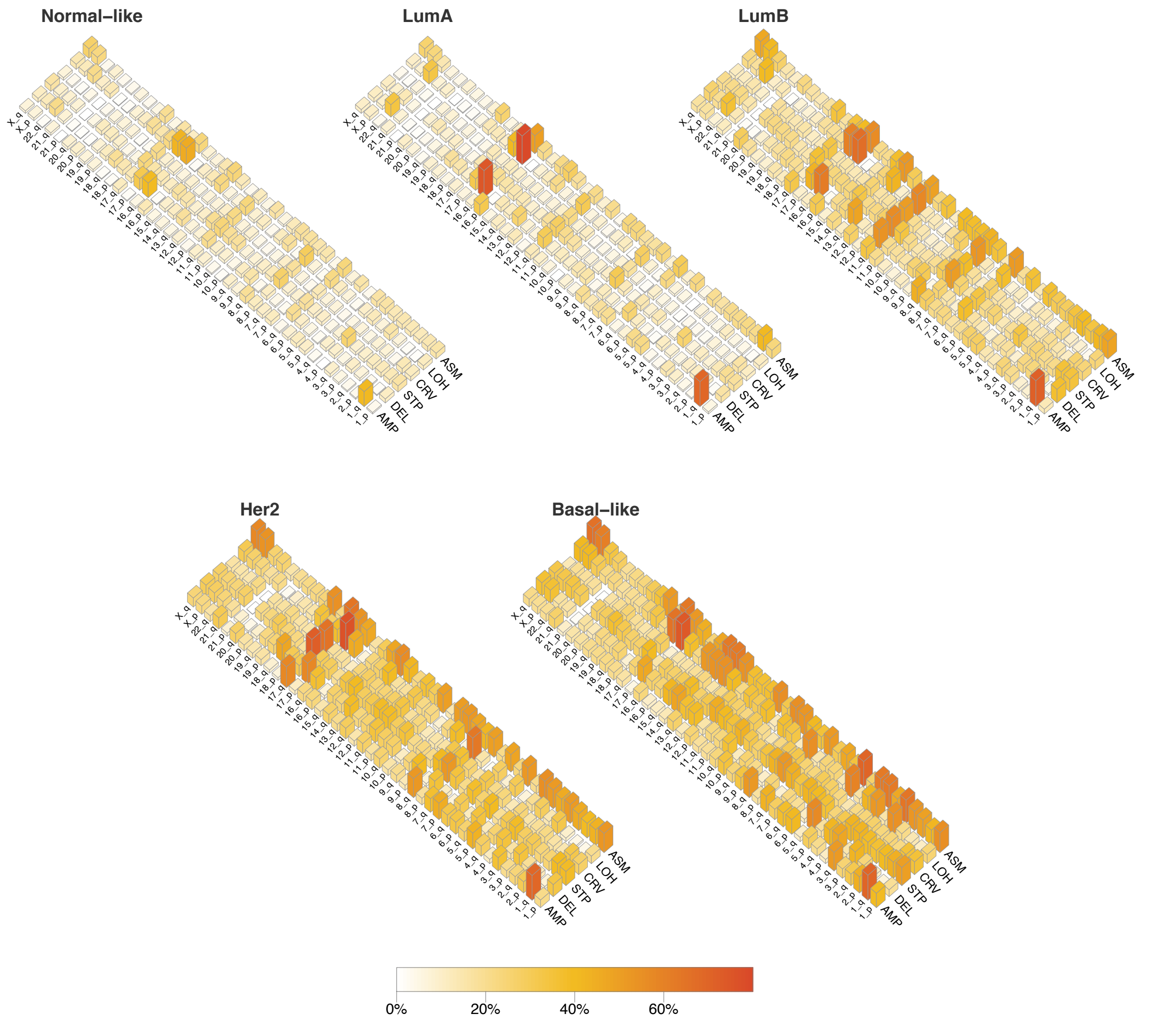

Supplementary Figure 4. Score landscapes for PAM50 subtypes in the METABRIC cohort. The bars reflect, for each of the six CARMA indices and each chromosome arm, the percentage of tumors in the METABRIC set that have a score larger than the index median (calculated across all arms and ignoring zero scores within each of the CARMA indices in the METABRIC discovery set).

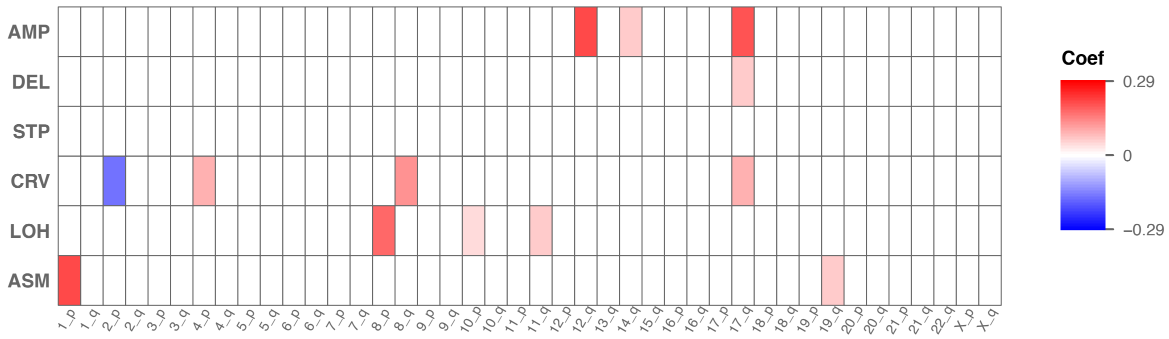

Supplementary Figure 5. Heatmap giving arm-specific coefficients from Lasso-Cox analysis of disease specific survival in the METABRIC discovery set.

Supplementary Tables

Score	HR	Lower 95% CI	Upper 95% CI	Z-score	P-value
AMP	5.66	3.61	8.89	7.54	5e-14
DEL	2.82	1.91	4.16	5.23	2e-07
STP	4.18	3.06	5.70	8.99	1e-19
CRV	3.36	2.58	4.38	8.95	1e-19
LOH	3.64	2.32	5.70	5.63	2e-08
ASM	8.22	4.68	14.46	7.31	4e-13
GII	2.92	2.22	3.84	7.67	1e-14

Supplementary Table 1. Univariate Cox regression on the six whole-genome CARMA scores found by unweighted averaging of the arm-wise scores. Results are shown for disease specific survival (DSS) in the METABRIC cohort ($n = 1943$). Shown is the estimated hazard ratio, 95 % confidence intervals for the hazard ratio, Z-score and P-value. Results are also shown for a model using the Genomic Instability Index (GII) as predictor.

Type	Covariate	P value	HR	Lower 95% CI	Upper 95% CI
Unadjusted	Age (≤ 60 vs > 60 years)	2.2e-01	1.19	0.91	1.55
Unadjusted	T-status (pT1, pT2, $\geq$pT3)	9.2e-07	1.76	1.40	2.20
Unadjusted	N-status (Positive vs Negative)	5.5e-09	2.28	1.73	3.01
Unadjusted	Histological grade (1-3)	5.9e-08	2.01	1.56	2.58
Unadjusted	Estrogen Receptor (Negative vs Positive)	2.0e-06	2.00	1.50	2.66
Unadjusted	HER2 receptor (Positive vs Negative)	1.6e-09	2.79	2.00	3.89
Unadjusted	Tp53 status (Mutated vs Wildtype)	7.2e-08	2.44	1.77	3.38
Unadjusted	GII ($+1SD$)	1.1e-09	1.52	1.33	1.73
Unadjusted	CPI (Low,Interm.,High risk)	1.9e-13	1.96	1.64	2.34
Unadjusted	CPI weighted (Low,Interm.,High risk)	5.2e-10	1.74	1.46	2.07
Adjusted	CPI (Low,Interm.,High risk)	3.4e-13	1.95	1.63	2.33
	Age (≤ 60 vs > 60 years)	5.6e-01	1.08	0.83	1.42
Adjusted	CPI (Low,Interm.,High risk)	5.5e-12	1.89	1.58	2.26
	T-status (pT1, pT2, $\geq$pT3)	1.1e-05	1.68	1.33	2.11
Adjusted	CPI (Low,Interm.,High risk)	5.6e-13	1.94	1.62	2.33
	N-status (Positive vs Negative)	1.8e-08	2.22	1.68	2.93
Adjusted	CPI (Low,Interm.,High risk)	2.2e-08	1.73	1.43	2.09
	Histological grade (1-3)	1.5e-03	1.54	1.18	2.01
Adjusted	CPI (Low,Interm.,High risk)	2.0e-11	1.87	1.56	2.25
	Estrogen Receptor (Negative vs Positive)	1.2e-03	1.62	1.21	2.16
Adjusted	CPI (Low,Interm.,High risk)	6.7e-10	1.80	1.49	2.17
	HER2 receptor (Positive vs Negative)	4.6e-04	1.87	1.32	2.65
Adjusted	CPI (Low,Interm.,High risk)	3.1e-05	1.64	1.30	2.08
	Tp53 status (Mutated vs Wildtype)	3.1e-03	1.72	1.20	2.46
Adjusted	CPI (Low,Interm.,High risk)	1.2e-06	1.76	1.40	2.21
	GII ($+1SD$)	1.3e-01	1.14	0.96	1.36
Adjusted	CPI weighted (Low,Interm.,High risk)	1.0e-09	1.73	1.45	2.07
	Age (≤ 60 vs > 60 years)	7.1e-01	1.05	0.80	1.38
Adjusted	CPI weighted (Low,Interm.,High risk)	6.7e-09	1.69	1.42	2.02
	T-status (pT1, pT2, $\geq$pT3)	8.8e-06	1.69	1.34	2.13
Adjusted	CPI weighted (Low,Interm.,High risk)	2.4e-09	1.71	1.43	2.04
	N-status (Positive vs Negative)	2.7e-08	2.20	1.66	2.90
Adjusted	CPI weighted (Low,Interm.,High risk)	6.4e-05	1.48	1.22	1.80
	Histological grade (1-3)	6.3e-04	1.61	1.22	2.11
Adjusted	CPI weighted (Low,Interm.,High risk)	1.0e-07	1.64	1.37	1.97
	Estrogen Receptor (Negative vs Positive)	2.0e-03	1.59	1.19	2.14
Adjusted	CPI weighted (Low,Interm.,High risk)	1.3e-06	1.57	1.31	1.89
	HER2 receptor (Positive vs Negative)	1.5e-04	1.99	1.39	2.84
Adjusted	CPI weighted (Low,Interm.,High risk)	6.0e-03	1.38	1.10	1.73
	Tp53 status (Mutated vs Wildtype)	3.6e-04	1.93	1.35	2.77
Adjusted	CPI weighted (Low,Interm.,High risk)	2.0e-03	1.45	1.14	1.83
	GII ($+1SD$)	1.7e-02	1.25	1.04	1.50

Supplementary Table 2. Prognostic value of clinical variables, CPI and $\text{CPI}_{weighted}$, and the genomic instability index (GII) for disease specific survival (DSS) in the METABRIC test set ($n = 648$). HR: hazard ratio. CI: confidence interval.

Type	Covariate	P value	HR	Lower 95% CI	Upper 95% CI
Unadjusted	Age ($\leq$ 60 vs $>$ 60 years)	5.3e-02	1.29	1.0	1.68
Unadjusted	T-status (pT1, pT2, $\geq$pT3)	2.9e-05	1.59	1.3	1.98
Unadjusted	N-status (Positive vs Negative)	8.2e-07	1.94	1.5	2.52
Unadjusted	Histological grade (1-3)	3.2e-06	1.74	1.4	2.20
Unadjusted	Estrogen Receptor (Negative vs Positive)	2.3e-03	1.57	1.2	2.09
Unadjusted	HER2 receptor (Positive vs Negative)	7.0e-06	2.19	1.6	3.09
Unadjusted	Tp53 status (Mutated vs Wildtype)	1.0e-04	1.90	1.4	2.62
Unadjusted	GII ($+1SD$)	3.6e-08	1.43	1.3	1.63
Unadjusted	CPI (Low,Interm.,High risk)	5.7e-13	1.87	1.6	2.22
Unadjusted	CPI weighted (Low,Interm.,High risk)	3.7e-07	1.53	1.3	1.80
Adjusted	CPI (Low,Interm.,High risk)	1.5e-12	1.85	1.56	2.20
	Age ($\leq$ 60 vs $>$ 60 years)	1.8e-01	1.19	0.92	1.55
Adjusted	CPI (Low,Interm.,High risk)	9.0e-12	1.83	1.54	2.17
	T-status (pT1, pT2, $\geq$pT3)	4.6e-04	1.49	1.19	1.86
Adjusted	CPI (Low,Interm.,High risk)	1.9e-12	1.86	1.56	2.21
	N-status (Positive vs Negative)	3.1e-06	1.87	1.44	2.44
Adjusted	CPI (Low,Interm.,High risk)	4.2e-09	1.76	1.46	2.13
	Histological grade (1-3)	3.7e-02	1.30	1.02	1.67
Adjusted	CPI (Low,Interm.,High risk)	1.5e-11	1.83	1.54	2.18
	Estrogen Receptor (Negative vs Positive)	1.6e-01	1.23	0.92	1.66
Adjusted	CPI (Low,Interm.,High risk)	1.9e-10	1.78	1.49	2.13
	HER2 receptor (Positive vs Negative)	3.4e-02	1.47	1.03	2.11
Adjusted	CPI (Low,Interm.,High risk)	5.6e-06	1.69	1.35	2.12
	Tp53 status (Mutated vs Wildtype)	1.7e-01	1.28	0.90	1.83
Adjusted	CPI (Low,Interm.,High risk)	4.5e-07	1.81	1.44	2.28
	GII ($+1SD$)	6.6e-01	1.04	0.87	1.24
Adjusted	CPI weighted (Low,Interm.,High risk)	7.8e-07	1.51	1.28	1.78
	Age ($\leq$ 60 vs $>$ 60 years)	1.4e-01	1.22	0.94	1.58
Adjusted	CPI weighted (Low,Interm.,High risk)	5.4e-07	1.53	1.29	1.80
	T-status (pT1, pT2, $\geq$pT3)	6.6e-05	1.57	1.26	1.95
Adjusted	CPI weighted (Low,Interm.,High risk)	1.5e-06	1.50	1.27	1.76
	N-status (Positive vs Negative)	3.4e-06	1.87	1.44	2.43
Adjusted	CPI weighted (Low,Interm.,High risk)	1.4e-04	1.40	1.18	1.66
	Histological grade (1-3)	3.2e-04	1.55	1.22	1.97
Adjusted	CPI weighted (Low,Interm.,High risk)	1.5e-06	1.50	1.27	1.78
	Estrogen Receptor (Negative vs Positive)	1.5e-02	1.44	1.07	1.92
Adjusted	CPI weighted (Low,Interm.,High risk)	5.4e-06	1.47	1.24	1.73
	HER2 receptor (Positive vs Negative)	2.7e-04	1.91	1.35	2.70
Adjusted	CPI weighted (Low,Interm.,High risk)	9.4e-05	1.51	1.23	1.86
	Tp53 status (Mutated vs Wildtype)	7.9e-03	1.57	1.13	2.19
Adjusted	CPI weighted (Low,Interm.,High risk)	2.6e-03	1.32	1.10	1.59
	GII ($+1SD$)	3.3e-04	1.30	1.13	1.50

Supplementary Table 3. Prognostic value of clinical variables, CPI and $\text{CPI}_{weighted}$, and the genomic instability index (GII) for progression free survival (PFS) in the METABRIC test set ($n = 648$). HR: hazard ratio. CI: confidence interval.

	METABRIC discovery	METABRIC test	Oslo2	OsloVal	ICGC
No of samples	1295	648	276	147	553
PAM50 subtypes					
LumA	461 (35.6)	249 (38.4)	107 (38.8)	29 (19.7)	122 (22.1)
LumB	323 (24.9)	158 (24.4)	63 (22.8)	47 (32)	61 (11)
Basal-like	215 (16.6)	106 (16.4)	36 (13)	26 (17.7)	65 (11.8)
Her2-enriched	159 (12.3)	77 (11.9)	31 (11.2)	21 (14.3)	17 (3.1)
Normal-like	133 (10.3)	56 (8.6)	15 (5.4)	24 (16.3)	4 (0.7)
IntClust subtypes					
IntClust1	91 (7)	47 (7.3)	22 (8)	4 (2.7)	20 (3.6)
IntClust2	41 (3.2)	30 (4.6)	10 (3.6)	21 (14.3)	7 (1.3)
IntClust3	190 (14.7)	97 (15)	45 (16.3)	11 (7.5)	54 (9.8)
IntClust4	216 (16.7)	113 (17.4)	40 (14.5)	26 (17.7)	34 (6.1)
IntClust5	124 (9.6)	60 (9.3)	22 (8)	19 (12.9)	6 (1.1)
IntClust6	62 (4.8)	22 (3.4)	14 (5.1)	24 (16.3)	8 (1.4)
IntClust7	122 (9.4)	65 (10)	15 (5.4)	7 (4.8)	40 (7.2)
IntClust8	195 (15.1)	101 (15.6)	32 (11.6)	17 (11.6)	28 (5.1)
IntClust9	95 (7.3)	48 (7.4)	17 (6.2)	3 (2)	21 (3.8)
IntClust10	159 (12.3)	65 (10)	25 (9.1)	8 (5.4)	45 (8.1)

Supplementary Table 4. Subtype distribution within the five study cohorts (METABRIC discovery, METABRIC test, Oslo2, OsloVal and ICGC). Shown is the number and proportion of samples in each cohort that were assigned to each of the five PAM50 subtypes and each of the ten IntClust subtypes.

	METABRIC discovery	METABRIC test	Oslo2	OsloVal	ICGC
No. of samples	1295	648	276	147	553
Age at diagnosis (median)	62	61.2	56.8	59	55
T-status					
pT1	557 (43)	285 (44)	150 (54.3)	57 (38.8)	99 (17.9)
pT2	664 (51.3)	321 (49.5)	110 (39.9)	58 (39.5)	152 (27.5)
pT3	62 (4.8)	34 (5.2)	12 (4.3)	9 (6.1)	33 (6)
pT4	0 (0)	0 (0)	0 (0)	16 (10.9)	10 (1.8)
N-status					
pN-	674 (52)	343 (52.9)	172 (62.3)	81 (55.1)	139 (25.1)
pN+	621 (48)	305 (47.1)	104 (37.7)	58 (39.5)	145 (26.2)
Histological grade					
Grade 1	115 (8.9)	50 (7.7)	40 (14.5)	11 (7.5)	48 (8.7)
Grade 2	519 (40.1)	244 (37.7)	116 (42)	59 (40.1)	152 (27.5)
Grade 3	614 (47.4)	331 (51.1)	119 (43.1)	41 (27.9)	153 (27.7)
Estrogen receptor					
Positive	993 (76.7)	487 (75.2)	222 (80.4)	114 (77.6)	369 (66.7)
Negative	282 (21.8)	152 (23.5)	54 (19.6)	33 (22.4)	184 (33.3)
Progesterone receptor					
Positive	680 (52.5)	344 (53.1)	196 (71)	72 (49)	316 (57.1)
Negative	615 (47.5)	304 (46.9)	80 (29)	63 (42.9)	228 (41.2)
HER2 status					
Positive	164 (12.7)	76 (11.7)	26 (9.4)	-	-
Negative	1131 (87.3)	572 (88.3)	250 (90.6)	-	-
Tp53 status					
Wildtype	692 (53.4)	332 (51.2)	183 (66.3)	-	-
Mutation	265 (20.5)	125 (19.3)	91 (33)	-	-
Survival analyses events					
BC deaths	428 (33.1)	212 (32.7)	-	58 (39.5)	39 (7.1)
All deaths	752 (58.1)	375 (57.9)	-	104 (70.7)	61 (11)

Supplementary Table 5. Overview of clinical and pathological variables within the five study cohorts (METABRIC discovery, METABRIC test, Oslo2, OsloVal and ICGC). Survival data were not available for the Oslo2 cohort; HER2 status and Tp53 status was not available for the OsloVal cohort and was not included for the ICGC cohort.

Supplementary Material and Methods

Data material

Oslo2

A total of 333 patients from the Oslo2 cohort were included in this study. Of these 19 non-invasive were excluded, while 38 samples did not return an ASCAT solution. This left 276 samples for further analyses. The Oslo2 study is an ongoing study where consecutive patients with primary operable (cT1-cT2) breast cancer are included at collaborating hospitals in southeast Norway. Patients were included at the time of surgery after informed consent. All patients in this study were included at Oslo University Hospital.

As described in a previous work [1, 2], tumor material was fresh frozen at - 80° C after macroscopic evaluation of the surgical specimen by an experienced pathologist. The Regional Committee for Medical and Health Research Ethics for southeast Norway have approved the study (approval number 1.2006.1607, amendment 1.2007.1125). Clinical information was collected from hospital records and histopathological data from routine assessment of the tumor. An overview of clinical and pathological variables is given in Supplementary Table 1-2. Fresh frozen tumor was cut with scalpel. One piece of the tumor tissue was used for DNA isolation using the Maxwell® 16 instrument (Promega, USA) and the Maxwell® 16 tissue DNA Purification Kit (Promega).

DNA was isolated according to manufacturer's protocol. In brief, tumor tissue was transferred into the Maxwell cartridge cassettes predispensed with magnetic beads, lysis buffer, and wash buffers of isopropanol and ethanol. The isolation procedure is automated, starting with sample lysis and tissue homogenization, followed by bead isolation of DNA, and finally the washing steps. The DNA was eluted in 200-600 l TE buffer (PH 8.5). DNA is stored at - 20° C. DNA concentration and quality were measured using Nanodrop® ND-1000 (NanoDrop Technologies, USA), which determines the absorbance in the sample by spectrophotometer. 1.5μl of DNA in solution was used for measurement. The quality of the samples (260/280 absorbance) ranged from 1.33- 2.14 (mean 1.86), the 260/230 absorbances ranged from 0.06-2.39 (mean 1.79). Total RNA isolation was performed using TRIzol (Invitrogen, Life Technologies Corporation, CA, USA) as described previously [3]. Tumor DNA was hybridized to Affymetrix SNP 6.0 arrays per the manufacturer's instructions (Affymetrix, Santa Clara, CA) at AROS Applied Biotechnology (Aarhus, Denmark). Samples that met the quality control criteria established by AROS were subject to further in-house quality assessment.

The mRNA expression was determined by SurePrint G3 Human GE 8·60K one-color microarrays (Agilent, Santa Clara, CA, USA) according to manufacturer's protocol (One-Color Microarray-Based Gene Expression Analysis, Low Input Quick Amp Labeling, v.6.5, May 2010). 100ng of RNA per sample was amplified and hybridized on array. The array

includes 42,405 unique 60-mer probes, targeting 27,958 Entrez Gene RNAs and 7,419 lincRNAs. Scanning was performed using Agilent Scanner G2565A and AgilentG3_GX_1Color was used as profile. The signals were extracted using FeatureExtraction v.10.7.3.1 and protocol GE1_107_Sep09. The data was quantile normalized, hospital-centered and log2-transformed [1].

METABRIC

The Molecular Taxonomy of Breast Cancer International Consortium (METABRIC) cohort consists of 1980 patients with primary breast cancer [4]. Fresh frozen tumor tissue were collected from tumor banks in the UK (Nottingham, Addenbrooke's in Cambridge and Guys hospital in London) and Canada (Vancouver and Manitoba). Copy number (Affymetrix SNP 6.0) and gene expression (Illumina HT-12 v3 Expression Beadchip) were available and deposited to the European Genome-Phenome Archive (EGA, http://www.ebi.ac.uk/ega/) hosted by the European Bioinformatics Institute (EBI, Hinxton, UK) with accession number EGAS00000000083. The expression data were processed as described by [4] and the raw intensities were quantile normalized and log2-transformed.

A total of 30 samples were excluded due to unknown, pre-invasive or benign histology. 7 samples did not return an ASCAT solution, leaving 1943 samples for further analyses. A random partition of the dataset (2:1 randomization) into a discovery set (n=1295) and a test set (n=648) was performed.

Extensive clinical annotation is available for these patients. Median follow-up time for the full set is 7.3 years. Tumor size were categorized according to AJCC guidelines [5]. Estrogen receptor status (ER) by immunohistochemistry (IHC) was available for 1914 cases (98.5%), the 29 other samples were scored by the expression value of *ESR1* as described in [6]. HER2 status status by IHC was not available and hence scored by the expression value of *HER2* [6]. The clinical variables are presented in Supplementary Table 1-2.

OsloVal

The OsloVal cohort is an historic archive material from the Norwegian Radium Hospital, Oslo, Norway, where excess tumor material was stored after biochemical ER assay. For a detailed description of this data cohort, confer [7]. The Regional Committee for Medical and Health Research Ethics for southeast Norway have approved the study (approval number 2010/498). DNA and RNA were isolated from 184 samples and profiled on Affymetrix SNP 6.0 arrays and Illumina HT12 Bead Chip. Three samples were excluded due to unknown/uncertain histology and 16 samples did not have an ASCAT solution, leaving 165 samples for further analysis. Clinical and pathological variables are given in Supplementary Table 1-2. The original ER-status obtained for the OsloVal samples was not used, as it involved different methods being used on different samples. Instead, the ER-status was determined by considering the expression of ESR1. Examination of the distribution of ESR1 expression values across the cohort revealed two distinct peaks separated by a trough at

$ESR1 \approx 7.5$, and based on this we called tumors with $ESR1 < 7.5$ as ER-negative and tumors with $ESR1 \geq 7.5$ as ER-positive.

The copy number profiles of the samples in the OsloVal cohort were more affected by noise than the two other datasets, with numerous very short, scattered low-level copy number changes across the whole genome. As a consequence, we filtered out segments that were shorter than 5Mb and differed by no more than two copies from its neighboring segments. This approach proved to perform well for removing short spikes, while preserving more complex rearrangements like firestorms.

ICGC

This data set is described in detail in [8]. DNA was extracted from 560 breast cancers and normal tissue (peripheral blood lymphocytes, adjacent normal breast tissue or skin). Total RNA was extracted from 268 of these individuals. The included samples had previously been subjected to pathology review, and only samples with an estimated 70% or more of tumor cells were accepted for inclusion in the study. Two pathologists assessed paraffin-embedded and frozen sections for all samples where histological slides were available. Clinical data were recorded according to the proforma specified by the International Cancer Genome Consortium (ICGC) where possible. The finalized clinical and pathology data is provided as Supplementary Table 1 in [8]. Genome and transcriptome sequence data, and SNP6 array data, have been deposited at the European Genome-Phenome Archive (http://www.ebi.ac.uk/ega/ at the EBI) with accession number EGAS00001001178.

Single nucleotide polymorphism (SNP) array hybridization using the Affymetrix SNP6.0 platform was performed according to Affymetrix protocols. Estimates of copy number (CN) and B Allele Frequency (BAF) for breast cancer cases analyzed by Affymetrix 6.0 SNP microarrays were generated using CRMAv2 and ACNE in combination, as outlined in the ACNE vignette (http://www.aroma-project.org/). Estimates were made in an unmatched fashion using 270 HapMap cases obtained from Affymetrix (www.affymetrix.com) as a specific reference set in the NmfSnpPlm function, as SNP6 data from matched normal samples were not available. After estimation of CN and BAF values, 931,000 SNP probes were used for further analyses. All SNP probes were mapped to the hg19 genome build. Allele-specific copy number analysis of tumors was performed using ASCAT and was available for 553 tumors.

Regional instability score algorithm

We now describe the algorithm for computation of the six regional instability scores defined in the previous section. The region can be chosen as any contiguous section of a chromosome. In this paper, we focus on the case where regions correspond to chromosome arms. With 42 chromosome arms(excluding the short arms of the acrocentric chromosomes) we thus obtain $6 \times 42 = 252$ score values per tumor sample.

Suppose the genomic region has length L, and let the associated allele-specific copy number profile be (n_{Ai}, n_{Bi}), $i = 1, \ldots, m$. Let p_i be the physical location of the ith probe and $s_i = p_i/L$ the corresponding mapped location of the ith probe in $R = [0, 1]$. When-

ever $n_{Ak} + n_{Bk} \neq n_{A,k+1} + n_{B,k+1}$, we define $(s_k + s_{k+1})/2$ to be a change point. Let $0 < t_1 < t_2 < \cdots < t_{r-1} < 1$ be all the change points, and define $t_{-2} = t_{-1} = t_0 = 0$ and $t_r = t_{r+1} = t_{r+2} = 1$. The total copy number is then

$$f(t) = n_{Ak_i} + n_{Bk_i}, \quad t \in [t_{i-1}, t_i) \tag{1}$$

for $i = 1, \dots, r$, where k_i satisfies $s_{k_i} \in [t_{i-1}, t_i)$. In the following, the function $f(t)$ will be represented by the parameterization $f_0 = 0$, $f_i = f(t_{i-1})$ $(i = 1, \dots, r)$, $f_{r+1} = 0$. Now recall that $Df(t)$ is the slope of the line segment connecting the pair of segment centers immediately to the left and right of position t. Defining $t_i^{(1)} = (t_{i-1} + t_i)/2$ for $i = -1, 0, \dots, r+2$, we thus have

$$Df(t) = \frac{f_i - f_{i-1}}{t_i^{(1)} - t_{i-1}^{(1)}}, \quad t \in [t_{i-1}^{(1)}, t_i^{(1)}) \tag{2}$$

for $i = 1, \dots, r+1$. In the following, the function $Df(t)$ will be represented by the parameterization $(Df)_0 = 0$, $(Df)_i = Df(t_{i-1}^{(1)})$ $(i = 1, \dots, r+1)$, $(Df)_{r+2} = 0$. We define $D^2 f(t)$ in a similar manner by first letting $t_i^{(2)} = (t_{i-1}^{(1)} + t_i^{(1)})/2$ for $i = 0, \dots, r+1$, and

$$D^2 f(t) = \frac{(Df)_i - (Df)_{i-1}}{t_i^{(2)} - t_{i-1}^{(2)}}, \quad t \in [t_{i-1}^{(2)}, t_i^{(2)}) \tag{3}$$

for $i = 1, \dots, r+2$. In the following, the function $D^2 f(t)$ will be represented by the parameterization $(D^2 f)_i = D^2 f(t_{i-1}^{(2)})$ $(i = 1, \dots, r+1)$, $(D^2 f)_{r+2} = 0$.

Evaluation of the scores $J_1, \dots, J_6$ involves integration of piecewise constant functions, and can thus be expressed as finite sums over the segments.

We have

$$J_1 = \sum_{i=1}^{r} (t_i - t_{i-1})(f_i)_+^2 \tag{4}$$

$$J_2 = \sum_{i=1}^{r} (t_i - t_{i-1})(f_i)_-^2 \tag{5}$$

$$J_3 = \sum_{i=1}^{r+1} (t_i^{(1)} - t_{i-1}^{(1)})(Df)_i^2 = \sum_{i=1}^{r+1} \frac{(f_i - f_{i-1})^2}{t_i^{(1)} - t_{i-1}^{(1)}} \tag{6}$$

$$J_4 = \sum_{i=1}^{r+2} (t_i^{(2)} - t_{i-1}^{(2)})(D^2 f)_i^2 = \sum_{i=1}^{r+2} \frac{((Df)_i - (Df)_{i-1})^2}{t_i^{(2)} - t_{i-1}^{(2)}} \tag{7}$$

To insure robustness against outliers, $\psi_R^{(1)}$ and ψ were calculated as the median rather than the mean of the regional and genome-wide copy number distribution. In most cases, this will have a small effect, however, as it is our experience that the median is usually close to the mean for the type of data considered here. The scores in equations (6) and (7) are weighted by

the size of the genomic region. These scores capture focal complex events of a size that do not necessarily reflect the size of the genomic region, hence weighting is required to counteract the normalization of the genomic scale to the interval $[0, 1]$. In addition, to ensure that the values of these two indices are not unduly influenced by very small distances between break points, each term in the right hand sides of these equations is dampened by application of a soft threshold function $x \mapsto \tanh(x)$. Specifically, we have $J_2 = \sum (f_i - f_{i-1})^2 \cdot \tanh(1/(t_i^{(1)} - t_{i-1}^{(1)}))$ and $J_3 = \sum ((Df)_i - (Df)_{i-1})^2 \cdot \tanh(1/(t_i^{(2)} - t_{i-1}^{(2)}))$. Evaluation of J_6 requires allele-specific copy number values. Whenever $n_{Ak} \neq n_{A,k+1}$ or $n_{Bk} \neq n_{B,k+1}$, we define $(s_k + s_{k+1})/2$ to be a change point. Denoting change points $0 < t_1 < t_2 < \cdots < t_{r-1} < 1$, define $t_0 = 0$ and $t_r = 1$ and observe that the number of A-alleles and B-alleles is

$$f_A(t) = n_{Ak_i} \text{ and } f_B(t) = n_{Bk_i} \quad t \in [t_{i-1}, t_i) \tag{8}$$

for $i = 1, \ldots, r$, where k_i satisfies $s_{k_i} \in [t_{i-1}, t_i)$. Let $f_A(t)$ be represented by the parameterization $f_{A0} = 0$, $f_{Ai} = f_A(t_{i-1})$ $(i = 1, \ldots, r)$, $f_{A,r+1} = 0$, and let $f_B(t)$ be represented by $f_{B0} = 0$, $f_{Bi} = f_B(t_{i-1})$ $(i = 1, \ldots, r)$, $f_{B,r+1} = 0$. Then,

$$J_5 = \sum_{i=1}^{r} (t_i - t_{i-1}) 1_0(f_{Bi}) \tag{9}$$

$$J_6 = \sum_{i: f_{Ai}=0} (t_i - t_{i-1})(f_{Ai} - f_{Bi})^2 \tag{10}$$

Standardization of scores

All six scores were $\log_2$-transformed and normalized by dividing by the 99th percentile. To have a common reference, the 99th percentile in the METABRIC discovery set were used to standardize all data sets.

GII

The Genomic Instability Index (GII) is the fraction of the genome with aberrant copy number, here defined as an aberration from the median total copy number across the whole genome (denoted ψ). Formally, this can be expressed as $GII = \int_R I(|f(t) - \psi| > 0)\, dt$ where $f(t)$ denotes total copy number at locus $t \in R$, $I(.)$ takes values 0 and 1 depending on whether its argument is false or true, and $R = [0, 1]$ denotes positions on the genome. We applied GII to ASCAT processed copy number data where an estimate of the total number of DNA copies is provided for each segment. A bespoke implementation of the GII algorithm was used for this project (see the accompanying web site).

CINdex

CINdex is based on segmented copy number data and calculates a measure of the genomic instability across a chromosome (the method can also be applied at higher genomic resolutions). The method calls copy number values above some threshold t_{gain} as gains, and copy

number values below some threshold t_{loss} as losses. The amplitude of change is scaled in order to make maximal losses and maximal gains comparable in magnitude. CINdex was applied to ASCAT processed copy number data where an estimate of the total number of DNA copies is provided for each segment. A bespoke implementation of the CINdex algorithm was used for this project (see the accompanying web site).

GISTIC

GISTIC identifies genomic regions that are significantly amplified or deleted across multiple samples. Each aberration is given a score reflecting the aberration amplitude and the aberration frequency. Regions with false discovery rates below a given threshold are called as significant. For each significant region, the algorithm identifies a peak region, a wide peak region and region limits (of increasing genomic width). GISTIC produces multiple outputs, including a categorical value (0,1 or 2) of aberration for each region and each sample. In this paper, we have used this output and we only distinguish between value=0 and value¿0. CINdex was applied to PCF segmented copy number data using the parameter values $\gamma = 50$ and $k_{\text{min=5}}$ [9].

CAAI

The complex arm-wise aberration index (CAAI) was calculated as described in [10]. In brief, a score S_k was calculated for each chromosome arm $k = 1, \ldots, 42$ as described below, and then CAAI was defined as the maximum of these scores. Note that in this paper we use CAAI as a continuous-valued score, while in the original paper CAAI-values were thresholded at the value 0.5 to produce a dichotomous variable. To calculate the score S_k for arm k, the break points in f were first identified. For any given break point, let L_1 and L_2 denote the genomic size (in nucleotides) of the segments joined, let H_1 and H_2 denote their height (i.e. the total number of copies of DNA), and calculate $P = \tanh(\alpha/(L_1 + L_2))$, $Q = \tanh(\beta \cdot |H_1 - H_2|)$, and $W = 0.5(1 + \tanh(10P - 5)/\tanh(5))$. Here, $\alpha = 2 \cdot 10^6$ and $\beta = (1.2)^{-1}$. Next, for every window of size 20Mb calculate the sum of $W \cdot \min(P, Q)$ over all break points within the window. Then S_k is the maximal value of these sums.

Breast cancer subtyping

PAM50 subtypes were found using the method described in [11] and with the subtype centroids provided on the accompanying web site. In brief, for each sample, the gene expression values for the 50 genes in the PAM50 gene list were extracted. Three genes in the PAM50 signature were replaced with synonyms in our data, namely *CDCA1* with *NUF2*, *KNTC2* with *NDC80*, and *ORC6L* with *ORC6*. When multiple probes with identical PAM50 gene identifier was found, the average probe value was used for Agilent expression data and what was considered to be the probe least affected by variant splicing was used for Illumina expression data. It is recommended in [11] to perform an initial test-to-train set normalization, and for this purpose two centroids were calculated by averaging gene expression values over all

ER-positive samples and all ER-negative samples, respectively. Clinical ER status obtained through immunohistochemical staining of ER was used. A combined centroid was next defined as a weighted average of the ER-negative centroid and the ER-positive centroid, the weights being c and 1-c, where c is the proportion of ER-negative samples in the original data set (the training data set) used to define the PAM50 centroids. The samples to be subtyped were then centered by aligning the combined centroid with the centroid of the training data set, achieved by subtracting the combined centroid from the expression vector of each sample and then adding the centroid of the training data set. Finally, a subtype label (Luminal A, Luminal B, Her2-enriched, Basal-like, or Normal-like) was assigned to each sample by calculating the Spearman correlation between the sample's expression vector and each of the five PAM50 centroids and selecting the one with the highest correlation.

IntClust subtypes were determined by the approach described in [4], which is based on 754 features (39 segmented copy number features and 715 gene expression values). Features were first matched, using genomic position or gene name for copy number features and gene name for expression features. To make features comparable to those in the original dataset, they were normalized as outlined in [4]. Finally, samples were assigned to the 10 classes by the Nearest Shrunken Centroids method [12], using the original centroids and within-cluster standard deviations of each of the 754 features

Supplementary Table 1 provides an overview of the distribution of the PAM50 subtypes and the IntClust subtypes in each dataset.

References

[1] Aure, M.R., Vitelli, V., Jernström, S., Kumar, S., *et al.*: Integrative clustering reveals a novel split in the luminal A subtype of breast cancer with impact on outcome. Breast cancer research : BCR **19**(1), 44 (2017)

[2] Aure, M.R., Jernström, S., Krohn, M., Vollan, H.K.M., *et al.*: Integrated analysis reveals microRNA networks coordinately expressed with key proteins in breast cancer. Genome medicine **7**(1), 21 (2015)

[3] Sørlie, T., Wang, Y., Xiao, C., Johnsen, H., *et al.*: Distinct molecular mechanisms underlying clinically relevant subtypes of breast cancer: gene expression analyses across three different platforms. BMC Genomics **7**, 127 (2006)

[4] Curtis, C., Shah, S.P., Chin, S.F., Turashvili, G., *et al.*: The genomic and transcriptomic architecture of 2,000 breast tumours reveals novel subgroups. Nature **486**(7403), 346–352 (2012)

[5] Singletary, S.E., Allred, C., Ashley, P., Bassett, L.W., *et al.*: Revision of the American Joint Committee on Cancer staging system for breast cancer. J. Clin. Oncol. **20**(17), 3628–3636 (2002)

[6] Lehmann, B.D., Bauer, J.A., Chen, X., Sanders, M.E., *et al.*: Identification of human triple-negative breast cancer subtypes and preclinical models for selection of targeted therapies. J. Clin. Invest. **121**(7), 2750–2767 (2011)

[7] Margolin, A.A., Bilal, E., Huang, E., Norman, T.C., *et al.*: Systematic analysis of challenge-driven improvements in molecular prognostic models for breast cancer. Sci Transl Med **5**(181), 181–1 (2013)

[8] Nik-Zainal, S., Davies, H., Staaf, J., Ramakrishna, M., Glodzik, D., Zou, X., Martincorena, I., Alexandrov, L.B., Martin, S., Wedge, D.C., Van Loo, P., *et al.*: Landscape of somatic mutations in 560 breast cancer whole-genome sequences. Nature **534**(7605), 47–54 (2016). doi:10.1038/nature17676

[9] Nilsen, G., Liestøl, K., Van Loo, P., Vollan, H.K.M., *et al.*: Copynumber: Efficient algorithms for single- and multi-track copy number segmentation. BMC Genomics **13**, 591 (2012)

[10] Russnes, H.G., Vollan, H.K.M., Lingjærde, O.C., Krasnitz, A., *et al.*: Genomic architecture characterizes tumor progression paths and fate in breast cancer patients. Sci Transl Med **2**, 38–47 (2010)

[11] Parker, J.S., Mullins, M., Cheang, M.C.U., Leung, S., *et al.*: Supervised risk predictor of breast cancer based on intrinsic subtypes. Journal of Clinical Oncology **27**, 1160–1167 (2009)

[12] Tibshirani, R., Hastie, T., Narasimhan, B., Chu, G.: Diagnosis of multiple cancer types by shrunken centroids of gene expression. Proc. Natl. Acad. Sci. U.S.A. **99**(10), 6567–6572 (2002)
