# Supplementary material for: DNA copy number motifs are strong and independent predictors of survival in breast cancer
Source: Commun Biol. 2020 Apr 2;3:153. doi: 10.1038/s42003-020-0884-6 (PMC7118095; doi:10.1038/s42003-020-0884-6)
Supplement: Supplementary file 2 — Description of Additional Supplementary Files [file 42003_2020_884_MOESM2_ESM.pdf]

Description of Additional Supplementary Files

File Name: **Supplementary Data 1**
Description:

- Segmented allele specific copy number data and regional CARMA scores for the three samples OSL1, OSL2 and OSL3 displayed in Figure 1 e.
- Segmented allele specific copy number data, regional CARMA, CAAI and CINdex scores in addition to GISTIC amp and del regions for the samples MB-0010 and MB-0028 displayed in Figure 2.
- Frequency tables of CARMA scores for each IntClust subtype in the METABRIC cohort as displayed in Figure 3 a.
- Numerical scores for the CARMA scores AMP, CRV and LOH as well as Pam50 subtyping for tumors in the OSLO2 and METABRIC cohort as displayed in Figure 3 b.
- Survival data and CPI stratification for the three validation cohorts OsloVal, BASIS and METABRIC test as displayed in Figure 3 e
