# Supplementary material for: DNA copy number motifs are strong and independent predictors of survival in breast cancer
Source: Commun Biol. 2020 Apr 2;3:153. doi: 10.1038/s42003-020-0884-6 (PMC7118095; doi:10.1038/s42003-020-0884-6)
Supplement: Supplementary file 4 — Reporting Summary [file 42003_2020_884_MOESM4_ESM.pdf]

nature research

Corresponding author(s): Ole Christian Lingjærde

Last updated by author(s): Feb 27, 2020

Reporting Summary

Nature Research wishes to improve the reproducibility of the work that we publish. This form provides structure for consistency and transparency in reporting. For further information on Nature Research policies, see Authors & Referees and the Editorial Policy Checklist.

Statistics

For all statistical analyses, confirm that the following items are present in the figure legend, table legend, main text, or Methods section.

n/a	Confirmed	
☐	☒	The exact sample size (*n*) for each experimental group/condition, given as a discrete number and unit of measurement
☐	☒	A statement on whether measurements were taken from distinct samples or whether the same sample was measured repeatedly
☐	☒	The statistical test(s) used AND whether they are one- or two-sided<br>*Only common tests should be described solely by name; describe more complex techniques in the Methods section.*
☐	☒	A description of all covariates tested
☐	☒	A description of any assumptions or corrections, such as tests of normality and adjustment for multiple comparisons
☐	☒	A full description of the statistical parameters including central tendency (e.g. means) or other basic estimates (e.g. regression coefficient) AND variation (e.g. standard deviation) or associated estimates of uncertainty (e.g. confidence intervals)
☐	☒	For null hypothesis testing, the test statistic (e.g. *F*, *t*, *r*) with confidence intervals, effect sizes, degrees of freedom and *P* value noted<br>*Give P values as exact values whenever suitable.*
☐	☒	For Bayesian analysis, information on the choice of priors and Markov chain Monte Carlo settings
☐	☒	For hierarchical and complex designs, identification of the appropriate level for tests and full reporting of outcomes
☐	☒	Estimates of effect sizes (e.g. Cohen's *d*, Pearson's *r*), indicating how they were calculated

Our web collection on statistics for biologists contains articles on many of the points above.

Software and code

Policy information about availability of computer code

Data collection	No software was used except the built-in preprocessing software in the scanning instruments described in Supplementary Materials.
Data analysis	Copy number data:<br>PennCNV was used to preprocess Affymetrix CEL-files and to obtain LogR and B-allele frequency (BAF) values. ASCAT v2.3 was used to determine allele-specific copy numbers, tumor cell percentage and tumor ploidy. CARMA v1.0 was used to derive copy number motifs from the ASCAT profiles.<br>mRNA expression data:<br>mRNA signals were extracted using FeatureExtraction v.10.7.3.1 and protocol GE1 107 Sep09. The PAM50 algorithm and the IntClust algorithm were used to derive subtypes.<br>All analyses were performed in R version 3.3.3.

For manuscripts utilizing custom algorithms or software that are central to the research but not yet described in published literature, software must be made available to editors/reviewers. We strongly encourage code deposition in a community repository (e.g. GitHub). See the Nature Research guidelines for submitting code & software for further information.

Data

Policy information about availability of data

All manuscripts must include a data availability statement. This statement should provide the following information, where applicable:

- Accession codes, unique identifiers, or web links for publicly available datasets
- A list of figures that have associated raw data
- A description of any restrictions on data availability

Copy number, gene-expression and clinical data for the OsloVal cohort are available at the Synapse platform, DOI: 10.7303/syn1688370. Gene-expression data for the Oslo2 cohort are available at Gene Expression Omnibus, DOI: GSE81002. Copy number data and subtypes for the Oslo2 cohort are available upon request. Copy number and expression data are available for the Metabric cohort at European Genome-Phenome Archive, EGAC00001000484. Copy number, gene expression and

clinical data are available for the ICGC cohort in the Supplementary Information of Nik-Zainal et al., Landscape of somatic mutations in 560 breast cancer whole-genome sequences, Nature, Vol 534, 2016. Raw data are also available from the European Genome-Phenome Archive under the accession number EGAS00001001178.

Field-specific reporting

Please select the one below that is the best fit for your research. If you are not sure, read the appropriate sections before making your selection.

☒ Life sciences ☐ Behavioural & social sciences ☐ Ecological, evolutionary & environmental sciences

For a reference copy of the document with all sections, see nature.com/documents/nr-reporting-summary-flat.pdf

Life sciences study design

All studies must disclose on these points even when the disclosure is negative.

Sample size	Sample sizes were chosen on the basis of availability of samples within each respective cohort.
Data exclusions	In the Oslo2 cohort, 19 samples were excluded because they were not invasive cancers and 38 additional samples were excluded because the did not return an ASCAT solution. In the Metabric cohort, a total of 30 samples were excluded due to unknown, pre-invasive or benign histology. In addition, 7 samples were excluded because ASCAT did not return a solution. In the OsloVal cohort, three samples were excluded due to unknown or uncertain histology, and 16 additional samples were excluded because ASCAT did not return a solution.
Replication	Validation was performed in four different cohorts (Metabric validation cohort, Oslo2 cohort , OsloVal cohort and ICGC cohort) and demonstrated that the findings in the paper were reproducible.
Randomization	The training data set was found through randomization of the samples in the Metabric cohort into a training data set (2/3 of the samples) and a test cohort (1/3 of the samples).
Blinding	Blinding was not relevant for this retrospective study. However, the validation was performed after the training session had been finalized and did not influence the final predictors.

Reporting for specific materials, systems and methods

We require information from authors about some types of materials, experimental systems and methods used in many studies. Here, indicate whether each material, system or method listed is relevant to your study. If you are not sure if a list item applies to your research, read the appropriate section before selecting a response.

Materials & experimental systems

n/a	Involved in the study	
☒	☐	Antibodies
☒	☐	Eukaryotic cell lines
☒	☐	Palaeontology
☒	☐	Animals and other organisms
☐	☒	Human research participants
☐	☒	Clinical data

Methods

n/a	Involved in the study	
☒	☐	ChIP-seq
☒	☐	Flow cytometry
☒	☐	MRI-based neuroimaging

Human research participants

Policy information about studies involving human research participants

Population characteristics	Population characteristics for the four study cohorts are described in detail in the Data Material section in Supplementary Materials and in the references therein.
Recruitment	Patient recruitment in the four study cohorts are described in detail in the Data Material section in Supplementary Materials and in the references therein.
Ethics oversight	For the Oslo2 cohort, the regional committee for medical and health research ethics for southeast Norway. For the OsloVal cohort, the regional committee for medical and health research ethics for southeast Norway.For the Metabric cohort. the ethics committees in Cambridge, UK, and Vancouver, Canada, the two sites responsible for the molecular analysis of the samples. For the ICGC cohort, internal review boards of each participating institution approved collection and use of samples of all patients in the study (for more details about this multi-center study, see Supplementary Methods and Data in Nik-Zainal et al, Landscape of somatic mutation in 560 breast cancer whole-genome sequences, Nature, 2016).

Note that full information on the approval of the study protocol must also be provided in the manuscript.

Clinical data

Policy information about clinical studies
All manuscripts should comply with the ICMJE guidelines for publication of clinical research and a completed CONSORT checklist must be included with all submissions.

Clinical trial registration	Oslo2 cohort: approval number 1.2006.1607, amendment 1.2007.1125 (The regional committee for medical and health research ethics for southeast Norway). OsloVal cohort: approval number 2010/498 (The regional committee for medical and health research ethics for southeast Norway). Metabric cohort: material was collected from biobanks in Cambridge, UK, and Vancouver, Canada; see Curtis et al (Nature, 2012) for full details. ICGC cohort: material was collected from biobanks in the participating institutions; see Nik-Zainal et al (Nature, 2016) for full details.
Study protocol	Study protocols for Oslo2 and OsloVal can be obtained from The regional committee for medical and health research ethics for southeast Norway.
Data collection	This is described in detail in the Data Material section in Supplementary Materials and in the references therein.
Outcomes	Not relevant for this study.
